# Significant Association of PD-L1 With CD44 Expression and Patient Survival: Avenues for Immunotherapy and Cancer Stem Cells Downregulation in Pancreatic Cancers

**DOI:** 10.1155/2024/3448648

**Published:** 2024-08-07

**Authors:** Saleema Mehboob Ali, Yumna Adnan, Zubair Ahmad, Tabish Chawla, S. M. Adnan Ali

**Affiliations:** ^1^ Department of Surgery Aga Khan University Hospital, Karachi, Pakistan; ^2^ Consultant Histopathologist Sultan Qaboos Comprehensive Cancer Care and Research Centre, Seeb, Oman; ^3^ Department of Pathology and Laboratory Medicine Aga Khan University Hospital, Karachi, Pakistan

**Keywords:** cancer stem cell markers, immune checkpoint inhibitor, immunohistochemistry, pancreatic neoplasms

## Abstract

**Background:** Pancreatic cancers are known for their aggressive nature. This aggressiveness may be attributed to the presence of cancer stem cells (CSCs), which promote relapse, metastasis, and resistance to chemotherapy. Targeting CSCs is essential to reverse this aggressiveness in pancreatic malignancies. Literature highlights the association of PD-L1 expression with CSCs in various cancers, suggesting immunotherapy as a promising therapeutic approach. This study is aimed at investigating the potential of immunotherapy in pancreatic cancers by examining its association with selected CSC marker expression.

**Method:** A retrospective cohort study was conducted involving 56 patients with confirmed diagnoses of pancreatic cancers at Aga Khan University Hospital from January 2015 to October 2022. After exclusions, based on refusal to provide consent or incomplete follow-up data, 38 patients were enrolled in the study. Immunohistochemistry was performed on formalin-fixed paraffin-embedded (FFPE) tumor tissue samples to assess the expression of CSC markers (CD133, CD44, and L1CAM) and immune checkpoint inhibitor marker (PD-L1). Statistical analysis was employed to determine associations between marker expression, clinical factors, and overall survival.

**Results:** The study revealed that 86.8% of pancreatic cancer cases exhibited positive PD-L1 expression. Moreover, a significant association of PD-L1 expression was observed with the presence of CD44 protein (*p* = 0.030), as well as with the overall survival of patients (*p* = 0.023).

**Conclusion:** Our findings show a significant association of PD-L1 with CD44 marker expression as well as patient survival. This research shows the potential to serve as the foundation for investigating the efficacy of immunotherapy in reducing CD44-expressing CSCs in pancreatic cancer, potentially enhancing patient outcomes.

## 1. Introduction

According to the World Health Organization (WHO), pancreatic neuroendocrine tumors (PNETs) and solid pseudopapillary neoplasm (SPPN) of the pancreas are rare histological subtypes of pancreatic tumors, comprising approximately 2% of the incidence burden [1–3]. PNETs are characterized by cystic/solid or mixed lesions while SPPNs are presented as capsules with cystic or solid components filled in [4]. The first-hand treatment option for both tumors is surgical resection. Postresection, favorable prognoses have been documented for both, with reported 5-year survival rates reaching 90%. However, due to diagnostic challenges, approximately 60% and 13% of all PNETs and SPPNs, respectively, are clinically presented with invasion/metastasis, rendering them unresectable [2, 5]. Unresectable tumors are treated with chemotherapy and radiotherapy regimes [6]. Furthermore, 17% and 10% of unresectable PNETs and SPPNs, respectively, are also associated with tumor recurrence [7, 8]. Due to extremely low incidence and favorable outcomes, PNET and SPPN remained understudied over the years. However, due to increasing cases of tumor malignancy and recurrence, it has become essential to comprehensively investigate underlying mechanisms to elucidate new treatment modalities.

One of the plausible explanations for invasion/metastasis and recurrence can be the presence of cancer stem cells (CSCs) [9]. The existence of CSCs is considered a key factor contributing to cancer relapse and metastasis, given their resistance to most chemotherapy treatments [10]. CSC markers like CD44, CD133, and L1CAM are considered to be poor prognostic indicators in various cancers [9, 11–13]. Studies have also shown high expression of PD-L1 on the surface of CSCs isolated from colon cancer and breast cancer [9]. PD-L1 protein expression is known to be exploited by tumor cells to employ immunosuppressive mechanisms to resist the antitumor response [14]. This further strengthens the hypothesis that CSCs can be the key players in the invasion/metastasis and recurrence of the tumors. The current study is aimed at bridging this knowledge gap by investigating the expression and association of selected CSC markers (CD44, CD133, and L1CAM) with an immune checkpoint inhibitor (PD-L1) in PNET and SPPN patients from the Pakistani population.

## 2. Materials and Methods

### 2.1. Patients

A retrospective cohort study was performed. A total of 56 patients with confirmed diagnoses of pancreatic cancer sub-types PNET or SPPN treated at Aga Khan University Hospital (AKUH), Karachi, Pakistan, from January 2015 to December 2022 were approached. Among these, those who either declined to provide consent, were younger than 18 years of age, or had incomplete follow-up data were excluded. Subsequently, the remaining 38 patients were enrolled for the study. All pancreatic tumor types other than PNET or SPPN were excluded. No gender-related criterion was set during the patient enrollment process. For each patient, informed consent was obtained. Patient demographics and clinical data were retrieved from hospital medical records. Telephonic interviews were conducted in order to evaluate health status. The study was performed in line with the Declaration of Helsinki and was approved by the Ethical Review Committee of the Aga Khan University Hospital.

### 2.2. Immunohistochemistry

Formalin-fixed paraffin-embedded (FFPE) tumor tissue blocks were obtained from the hospital's histopathology department. Representative blocks were selected for each patient and subjected to hematoxylin and eosin (H&E) staining by a senior histopathologist to confirm the tumor type, diagnosis and to ensure the presence of suitable tumor content in the selected blocks.

Subsequently, 4 *μ*m-thick sections were cut from each FFPE block using a semiautomatic microtome. These freshly cut sections were then processed to remove wrinkles and placed onto pre-coated glass slides. Immunohistochemistry for selected proteins (CD133, CD44, L1CAM, and PD-L1) was carried out using the EnVision FLEX, High pH (Link) system, Dako, Denmark. This involved deparaffinization, rehydration, high pH antigen retrieval, blocking of endogenous peroxidase reaction, incubation with primary and secondary antibodies, and visualization of the antigen-antibody reaction using diaminobenzidine (DAB) chromogen. Hematoxylin was used for counterstaining, and coverslips were mounted using toluene-free mounting media.

The evaluation and scoring of immunohistochemical results was performed by two independent pathologists using a microscope (magnification 20–40x). Scoring criteria were established for each antibody, and discrepancies between the observers were resolved using a conference microscope. Both negative and positive controls were included in each batch of the assay for result verification. Expression locations of all the selected antibodies were membrane and/or cytoplasm. Experimental conditions for each antibody were adapted from previously published study [15] and are explained in Table 1.

### 2.3. Statistical Analysis

Statistical analysis was carried out using SPSS® version 23. Pearson's chi-square test or Fisher's exact test (as appropriate) were used to evaluate the association between categorical variables. The Kaplan–Meier and log-rank tests were used for survival analysis. *p* value < 0.05 was considered significant in all analyses.

## 3. Results

### 3.1. Patient Demographics and Clinicopathological Characteristics

Among the 38 patients included in the study, 55.2% were female, and 44.7% belonged to ≤ 40-year age group. The mean age of patients was 42.82 ± 17.043 (age range being 18–73 years). In the cohort, 23.6% of patients had tumors in the head region, 31.5% were diagnosed with tumors in the body/tail region, while for the remaining patients, the tumor site was unknown. Among the tumor types, 57.8% were PNETs, while the remaining were SPPNs. The majority of tumors were detected at Stage II (50%) and possessed well-differentiated histology (65.7%) (Table 2). Lymphovascular invasion was identified in 15.7% of patients, while perineural invasion was observed in 42.1% of patients within the cohort. Additionally, 18.4% of patients presented diabetes comorbidity, whereas 15.7% had a history of smoking and underwent chemotherapy treatment (both variables considered individually).

### 3.2. Immunohistochemical Staining and Association Analysis

CD44 protein was analyzed in high and low expression categories, where 34.2% of patients were presented with high CD44 expression. Positive expression for CD133, L1CAM, and PD-L1 were 52.6%, 57.8%, and 86.8%, respectively. Among the positive expression of CD133 and L1CAM protein, mild positive expression had the highest frequency in 14 and 19 patients, respectively, while for PD-L1, moderate positive expression was detected in most patients (14 patients).

**Figure 1:**
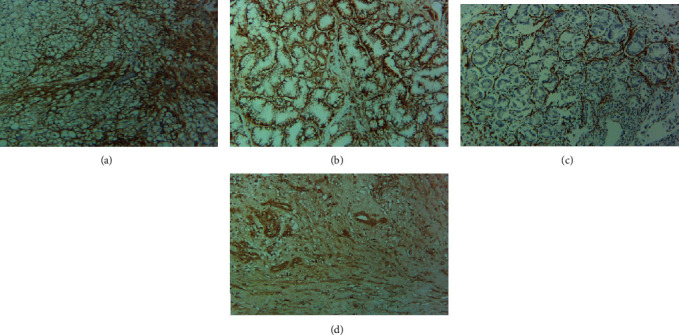
Immunohistochemical overexpression of the selected proteins in the study cohort (magnification—10x; scale—51 *μ*m): (a) CD133, (b) CD44, (c) L1CAM, and (d). PD-L1.

Details of immunohistochemical expression patterns of the proteins are described in Table 3. Positive immunohistochemical staining for all biomarkers is shown in Figure 1.

The Pearson chi-square test was used to assess the association between the expression of individual proteins and patient age group, gender, histological differentiation, and tumor type. As shown in Table 4, CD44 expression was significantly associated with PD-L1 expression (*p* = 0.030, OR: 9.429, 95% CI: 1.058–84.037) and marginally associated with PNET tumor type (*p* = 0.053, OR: 4.242, 95% CI: 0.935–19.256). On the other hand, CD133 expression was significantly associated with CD44 expression (*p* = 0.004, OR: 9.778, 95% CI: 1.762–54.263), L1CAM expression (*p* = 0.024, OR: 4.714, 95% CI: 1.178–18.861) and marginally associated with moderately differentiated tumor histology.

### 3.3. Association With Overall Survival (OS)

**Figure 2:**
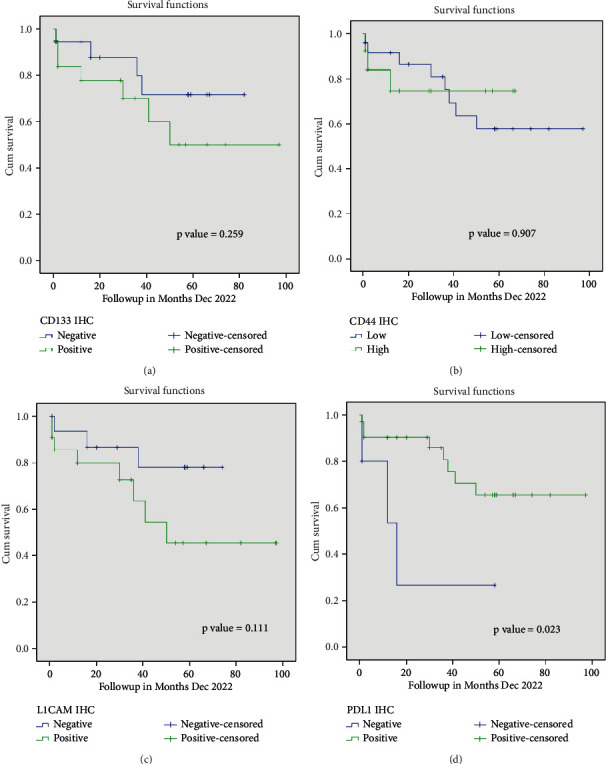
Kaplan–Meier curves for the association of protein biomarkers with overall survival of the patients: (a) CD133 expression, (b) CD44 expression, (c) L1CAM expression, and (d) PD-L1 expression.

The mean OS of the study patients was 34.55 ± 27.608 (1–97 months). A total of 11 patients (28.9%) died during the study period. Using the Kaplan–Meier and log-rank test, the association of OS was evaluated with expression of individual markers, staining intensity of the individual markers, and clinicopathological characteristics. As shown in Table 5 and Figure 2, the expression of PD-L1 was significantly associated with OS of the patient (*p* = 0.023). The mean survival duration for negative and positive expression of PD-L1 was 23.133 months and 73.463 months, respectively. Neither the expression/staining intensity of CSC markers nor tumor clinicopathological characteristics were found to possess any significant association with OS.

## 4. Discussion

In the present study, we evaluated the expression of selected CSC markers (CD133, CD44, and L1CAM) with immune checkpoint inhibitor (PD-L1) in pancreatic cancers. In our study, PD-L1 showed the highest positive expression (86.8%) as compared to any other biomarker. Other studies in the literature have reported contrasting expression levels ranging from 45% [16] to 73% [12]. Moreover, we also found a significant association between the expression of CD44 and PD-L1 (*p* = 0.030). These results are consistent with previous studies on breast cancers and colon cancers [9, 17]. Another study found significant expression of PD-L1 in ovarian CSCs expressing CD44 and LGR5 [11]. Moreover, in our cohort, PD-L1 expression was also shown to be significantly associated with OS of the patient. Similar results have been reported by earlier studies on pancreatic cancers (*p* = 0.013) [18]. These findings may have a significant impact on the patient treatment as immunotherapeutic agents targeting PD-1/PD-L1 are available. Target drugs for PD-L1—pembrolizumab and nivolumab—have passed Phase II clinical trials for use in PNETs. Apart from these, there are several PD-L1 immune checkpoint inhibitors approved by the Food and Drug Administration (FDA) in the USA for various cancers, for instance, atezolizumab for urothelial carcinoma and non-small cell lung carcinoma, ipilimumab for melanoma, and avelumab for Merkel cell carcinoma and urothelial carcinoma [19–21].

Environmental mutagens are well known for their role in carcinogenesis. These can be classified into physical, chemical, and biological categories. Physical mutagens include ionizing radiation and UV radiation, while chemical mutagens include heavy metals and organic compounds. Biological mutagens include pathogenic viruses and bacteria, such as *Helicobacter pylori*, which can induce DNA damage and contribute to cancer development [22]. Various environmental mutagens have been investigated for their role in aberrant protein expression in different cancers. For instance, tobacco smoke and related carcinogens, such as benzo[a]pyrene (BaP), may induce PD-L1 expression in lung cancers [23] and CD44 expression in head and neck cancers [24]. Epstein–Barr virus (EBV) infection is associated with PD-L1 overexpression in diffuse large B-cell lymphoma [25]. Hepatitis C virus infection has been shown to elevate CD44 expression in hepatocellular carcinoma [26]. Additionally, human papilloma virus (HPV) infection is linked to CD44 overexpression in oropharyngeal cancers [27], and *Helicobacter pylori* infections are associated with increased CD44 expression in colon cancers [28]. These studies highlight the role of infectious agents in elevated expression of PD-L1 and CD44 in cancer cells.

Another important finding from the current study is the significant association of CD44 with CD133 expression (*p* = 0.004) and CD133 with L1CAM expression (*p* = 0.024). This signals towards an underlying relationship between these three CSC markers. Moreover, the significance of these associations between CSC markers is underscored by the finding that CD44 is significantly associated with target immunotherapeutic marker PD-L1. Although conventional radiotherapy and chemotherapy offer therapeutic advantages against malignancies, clinical evidence suggests that CSCs exhibit resistance to these treatments. CSCs are recognized as a source of heterogeneity, recurrence, tumor progression, and therapy resistance. Hence, the identification of effective targeted therapies for CSCs would provide significant benefits to patients [29]. Previous investigations in literature have studied CSC markers CD44 and CD133 but did not report any association between their expressions [30]. The individual expression of L1CAM has been reported in a wide range—7% to 90% [31]. Further, an investigation evaluating the expression of L1CAM with any other CSC markers in pancreatic cancers was not found in the literature.

In our cohort, none of the clinicopathological characteristics was significantly associated with either protein expression or patient survival. However, the individual expression of CD133 and CD44 was found to be marginally associated with moderately differentiated histology of the tumor and PNET-type tumor, respectively. Literature shows varying trends of association analysis. CD44 was found to be significantly associated with high tumor grade, advanced stage, high mitotic count, poor tumor differentiation, and OS. On the other hand, CD133 did not show any association with clinicopathological characteristics but was highly associated with the OS [30].

In conclusion, this is the first study to investigate the association between CSC markers (CD133, CD44, and L1CAM) and the immune checkpoint inhibitor target biomarker (PD-L1) in PNETs and SPPN. Our findings show a significant association of PD-L1 with CD44 marker expression and patient survival. This research could lay grounds for evaluating the efficacy of immunotherapy to target CD44-expressing CSCs in pancreatic cancers, potentially improving patient outcomes. However, the current study has limitations, including a small sample size and a retrospective design. Future investigations with larger sample sizes and prospective designs are recommended to reinforce these findings. Additionally, molecular studies such as Western blotting and cell culture experiments should be conducted along with the inclusion of additional biomarkers (proteins and genes) in order to validate these preliminary results. Given the existing scientific literature also describes the role of tobacco and various viral infections in elevating PD-L1 and CD44 expression, these factors should also be investigated in future studies to obtain comprehensive insights.

## Figures and Tables

**Table 1:** Antibodies used for detection of target proteins included in the study.

**Antibody**	**Company**	**Clone**	**Antibody incubation time**	**Positive control**	**Dilution**	**Scoring criteria**
CD133	Abcam, UK	EPR16508	40 min	Known positive case of glioblastoma	1 : 1000	Negative (0) when no positive cells were observed within the tumor Mild (1) when < 30% of the tumor cells were positive Moderate (2) when 30%–60% of the tumor cells were positive Strong (3) when > 60% of tumor cells were positive

CD44	Dako, Denmark	DF1485	40 min	Known positive case of glioblastoma	1 : 40	0 = low or weak stained in 10% of cells or less; 1 = weak stained in 11–30% of cells; 2 = weak stained in more than 30% of cells or moderately stained in less than 30% of cells; 3 = moderate stained in 30%–60% of cells; 4 = moderate or strong stained in more than 60% of cells. The average staining scores were obtained based on 10 randomly selected fields in each slice. According to the above staining criteria, the scores (0–2) and (3–4) were regarded as low and high CD44 expression, respectively.

L1CAM	Abcam, UK	EPR18750	40 min	Normal kidney tissue	1 : 500	Intensity was defined as 0 = none, 1 = weak, 2 = moderate, and 3 = strong staining and multiplied with the percentage of positive cells including 0 = no cells, 1 = up to 10%, 2 = 10% to 50%, 3 = 51% to 80%, and 4 ≥ 80% with the intensity of staining assessed first. L1CAM expression was subsequently graduated in IRS scores 0 to 2 = negative, 3 to 4 = weakly positive, 6 to 8 = moderately positive, and 9 to 12 = strong.

PD-L1	Dako, Denmark	22C3	30 min	Known positive case of glioblastoma	1 : 50	Specimens with ≥ 10% PD-L1 positive tumor cells were classified as positive.

**Table 2:** Demographics and clinicopathological characteristics of the study cohort (*N* = 38, 100%).

**Variables**	**Frequency (%)**
*Gender*	
Male	17 (44.7%)
Female	21 (55.2%)
*Age group*	
≤ 40 years	17 (44.7%)
> 40 years	21 (55.2%)
*Tumor type*	
Neuroendocrine	22 (57.8%)
Solid pseudopapillary	16 (42.1%)
*Tumor site*	
Head	9 (23.6%)
Body/tail	12 (31.5%)
Specific site in pancreas unknown	17 (44.7%)
*T stage*	
T1	14 (36.8%)
T2	6 (15.7%)
T3	14 (36.8%)
T4	4 (10.5%)
*AJCC stage*	
I	19 (50%)
II	13 (34.2%)
III	6 (15.7%)
*Histological differentiation*	
Well	25 (65.7%)
Moderate	13 (34.2%)
Poor	0
*Health status*	
Alive	27 (71.05%)
Dead	11 (28.9%)
*Lymphovascular invasion*	
No	32 (84.2)
Yes	6 (15.7)
*Perineural invasion*	
No	22 (57.8)
Yes	16 (42.1)
*Diabetes*	
No	31 (81.5)
Yes	7 (18.4)
*Smoking*	
No	32 (84.2)
Yes	6 (15.7)
*Chemotherapy*	
No	32 (84.2)
Yes	6 (15.7)

**Table 3:** Protein expression profile of the cohort (*N* = 38, 100%).

**Protein**	**Frequency (%)**
*CD133*	
Positive expression	20 (52.6%)
Mild positive expression	14 (70%)^a^
Moderate positive expression	4 (20%)^a^
Strong positive expression	2 (10%)^a^
Negative expression	18 (47.3%)
*CD44*	
High expression	13 (34.2%)
Low expression	25 (65.7%)
*L1CAM*	
Positive expression	22 (57.8%)
Mild positive expression	19 (79.1%)^a^
Moderate positive expression	4 (16.6%)^a^
Strong positive expression	1 (4.1%)^a^
Negative expression	16 (42.1%)
*PD-L1*	
Positive expression	33 (86.8%)
Mild positive expression	11 (33.3%)^a^
Moderate positive expression	14 (42.4%)^a^
Strong positive expression	8 (24.2%)^a^
Negative expression	5 (13.1%)

^a^Percentages calculated taking the positive expression samples as total.

**Table 4:** Association between protein marker expression and selected demographical/clinicopathological features (chi-square/Fisher's exact test)—*N* = 38 (100%).

	**CD133**		**CD44**		**L1CAM**		**PD-L1**		**Gender**		**Age group**		**Histological differentiation**		**Tumor type**	
	**Negative^**	**Positive**	**Low^**	**High**	**Negative^**	**Positive**	**Negative^**	**Positive**	**Female^**	**Male**	**≤ 40 years^**	**> 40 years**	**Well^**	**Moderate**	**SPPN^**	**PNET**
CD133																
Negative	—	—	16 (64)	2 (15.4)	9 (36)	9 (69.2)	9 (36)	9 (69.2)	11 (52.4)	7 (41.2)	8 (47.1)	10 (47.6)	9 (36)	9 (69.2)	9 (52.9)	9 (42.9)
Positive	—	—	9 (36)	11 (84.6)	16 (64)	4 (30.8)	16 (64)	4 (30.8)	10 (47.6)	10 (58.8)	9 (52.9)	11 (52.4)	16 (64)	4 (30.8)	8 (47.1)	12 (57.1)
*p* value (odds ratio)	—		0.004^∗∗^ (9.778)		0.024^∗∗^ (4.714)		0.170 (5.429)		0.492 (1.571)		0.973 (0.978)		0.052^∗^ (4.000)		0.745 (1.500)	
CD44																
Low	16 (88.9)	9 (45)	—	—	15 (60)	10 (76.9)	4 (16)	21 (84)	14 (66.7)	11 (64.7)	12 (70.6)	13 (61.9)	15 (60)	10 (76.9)	14 (82.4)	11 (52.4)
High	2 (11)	11 (55)	—	—	10 (40)	3 (23.1)	1 (7.7)	12 (92.3)	7 (33.3)	6 (35.3)	5 (29.4)	8 (38.1)	10 (40)	3 (23.1)	3 (17.6)	10 (47.6)
*p* value (odds ratio)	0.004^∗∗^ (9.778)		—		0.743 (1.257)		0.030 (9.429)^∗∗^		0.899 (1.091)		0.575 (1.477)		0.473 (0.450)		0.053^∗^ (4.242)	
L1CAM																
Negative	11 (61.1)	5 (25)	11 (44)	5 (38.5)	9 (36)	7 (53.8)	9 (36)	7 (53.8)	8 (38.1)	8 (47.1)	8 (47.1)	8 (38.1)	9 (36)	7 (53.8)	9 (52.9)	7 (33.3)
Positive	7 (38.9)	15 (75)	14 (56)	8 (61.5)	16 (64)	6 (46.2)	16 (64)	6 (46.2)	13 (61.9)	9 (52.9)	9 (52.9)	13 (61.9)	16 (64)	6 (46.2)	8 (47.1)	14 (66.7)
*p* value (odds ratio)	0.024^∗∗^ (4.714)		0.743 (1.257)		—		1.000 (0.905)		0.578 (0.692)		0.578 (1.444)		0.290 (0.482)		0.224 (2.250)	
PD-L1																
Negative	4 (22.2)	1 (5)	4 (80)	1 (20)	2 (8)	3 (23.1)	2 (8)	3 (23.1)	3 (14.3)	2 (11.8)	2 (11.8)	3 (14.3)	2 (8)	3 (23.1)	3 (17.6)	2 (9.5)
Positive	14 (77.8)	19 (95)	21 (63.6)	12 (36.4)	23 (92)	10 (76.9)	23 (92)	10 (76.9)	18 (85.7)	15 (88.2)	15 (88.2)	18 (85.7)	23 (92)	10 (76.9)	14 (82.4)	19 (90.5)
*p* value (odds ratio)	0.117 (5.429)		0.030 (9.429)^∗∗^		1.000 (0.905)		—		1.000 (1.250)		1.000 (0.800)		0.315 (0.290)		0.640 (2.036)	

^^^Reference for odds ratio calculation.

^∗∗^Significant association (*p* < 0.05).

^∗^Marginal association (0.05 < *p* < 0.1).

**Table 5:** Association of patient demographics, tumor clinicopathological characteristics, and protein marker expression with overall survival of the patient—*N* = 38 (100%).

**Variables**	**Patients**	**Mean survival months**	**P** **value**
*Gender*			
Male	17	45.837	0.250
Female	21	77.311	
*Age group*			
≤ 40 years	17	73.168	0.674
> 40 years	21	57.313	
*Tumor type*			
Neuroendocrine	22	53.617	0.241
Solid pseudopapillary	16	77.524	
*Tumor site*			
Head	9	57.281	0.948
Body/tail	12	50.867	
Specific site in pancreas unknown	17	67.231	
*T stage*			
T1	14	58.093	0.514
T2	6	32.778	
T3	14	79.778	
T4	4	54	
*AJCC stage*			
I	19	56.878	0.190
II	13	87.50	
III	6	38.167	
*Histological differentiation*			
Well differentiated	25	72.452	0.477
Moderately differentiated	13	46.608	
Poorly differentiated	0	—	
*CD133 expression*			
Negative	18	65.872	0.259
Positive	20	60.970	
*CD44 expression*			
Low	25	68.192	0.907
High	13	51.340	
*L1CAM expression*			
Negative	16	62.213	0.111
Positive	22	58.597	
*PD-L1 expression*			
Negative	5	23.133	**0.023** ^∗^
Positive	33	73.463	
*Lymphovascular invasion*			
No	32	70.840	0.350
Yes	6	32.500	
*Perineural invasion*			
No	22	68.020	0.832
Yes	16	45.571	
*Diabetes*			
No	31	69.874	0.711
Yes	7	38.667	
*Smoking*			
No	32	75.279	0.370
Yes	6	32.889	
*Chemotherapy*			
No	32	66.600	0.564
Yes	6	69.833	

^∗^Significant association (*p* value < 0.05).

## Data Availability

The data used to support the findings of this study are included within the article.
